# Validity of tremor analysis using smartphone compatible computer vision frameworks

**DOI:** 10.1038/s41598-025-97252-4

**Published:** 2025-04-18

**Authors:** Robin Wolke, Julius Welzel, Walter Maetzler, Günther Deuschl, Jos Becktepe

**Affiliations:** Department of Neurology, UKSH, Kiel University, Kiel, Germany

**Keywords:** Neuroscience, Neurology, Movement disorders, Computational biology and bioinformatics

## Abstract

Computer vision (CV)-based approaches hold promising potential for the classification and quantitative assessment of movement disorders. To take full advantage of this potential, the pipelines need to be validated against established clinical and electrophysiological gold standards. This study examines the validity of the Mediapipe (by Google) and Vision (by Apple) smartphone-enabled hand detection frameworks for tremor analysis. Both frameworks were tested in virtual experiments with simulated tremulous hands to determine the optimal camera position for hand tremor assessment and the minimum detectable tremor amplitude and frequency. Both frameworks were then compared with optical motion capture (OMC), accelerometry, and clinical ratings in 20 tremor patients. Both CV frameworks accurately measured tremor peak frequency. Significant correlations were found between CV-assessed tremor amplitudes and Essential Tremor Rating Assessment Scale (TETRAS) scores. However, the accuracy of amplitude estimation compared to OMC as ground truth was insufficient for clinical application. In conclusion, CV-based tremor analysis is an accurate and simple clinical assessment tool to determine tremor frequency. Further improvements in amplitude estimation are needed.

## Introduction

As computer vision (CV) frameworks have become more sophisticated over the past decade, a growing number of medical professionals are interested in its application in movement disorders. When used correctly and focused on clinically relevant parameters, CV frameworks have the potential to add significant value to the assessment of movement disorders^1^.

One area of application could be the automatic quantification of tremor symptoms as described in Parkinson’s disease or essential tremor. However, the assessment of tremor as an oscillating, rhythmic movement presents with specific challenges. The upper limbs are the most common manifestation of tremor and the phenotypes range from variable amplitudes and frequencies (approximately 2–12 Hz), resting to action tremor and perfect sinusoidal to highly variable, irregular movements, making accurate clinical characterization difficult^2^. Optical motion capture (OMC) and accelerometry have been used to measure tremor parameters^3–6^. While the clinical application of OMC is limited due to significant cost, availability, and time, clinical observation combined with accelerometric and/or electromyographic tremor analysis are the usual tools to differentiate tremor and objectively measure frequency and amplitude^2^. However, polygraphic tremor analysis requires specialized hardware and knowledge typically available only to movement disorder specialists^7^. With the recent advances in computer vision algorithms, the potential of these algorithms for a wider range of tremor analysis applications remains to be explored.

Although several sophisticated approaches have been reported to analyze tremor in video recordings, they are still limited in their clinical applicability^3,8–13^. The current study evaluates the performance of two CV frameworks, Mediapipe (MP, Google) and Vision (VI, Apple), on tremor analysis^14,15^. We investigated the accuracy of amplitude and frequency estimation in comparison to optical motion capture (OMC) and 3-dimensional accelerometry using an inertial measurement unit (IMU). Both CV frameworks were of interest because they can be easily deployed on a smartphone. This is an advantage that lowers the threshold for clinical use.

However, to better understand the best processing pipeline for the video-based analysis of tremor parameters, we evaluated our approach on a synthetic dataset of hand tremor movements. By applying 3D motion simulation, influencing factors such as camera position or the tremor amplitude can be controlled for. The integration of synthetic data is commonly used for both evaluation and training of state-of-the-art computer vision algorithms and is also applied in medicine^16–19^. We then evaluated the performance of the CV frameworks on video data from patients with tremor, taking into account the prior knowledge derived from the virtual experiment for the experimental setup and data analysis.

## Results

### Virtual experiment

**Fig. 1:**
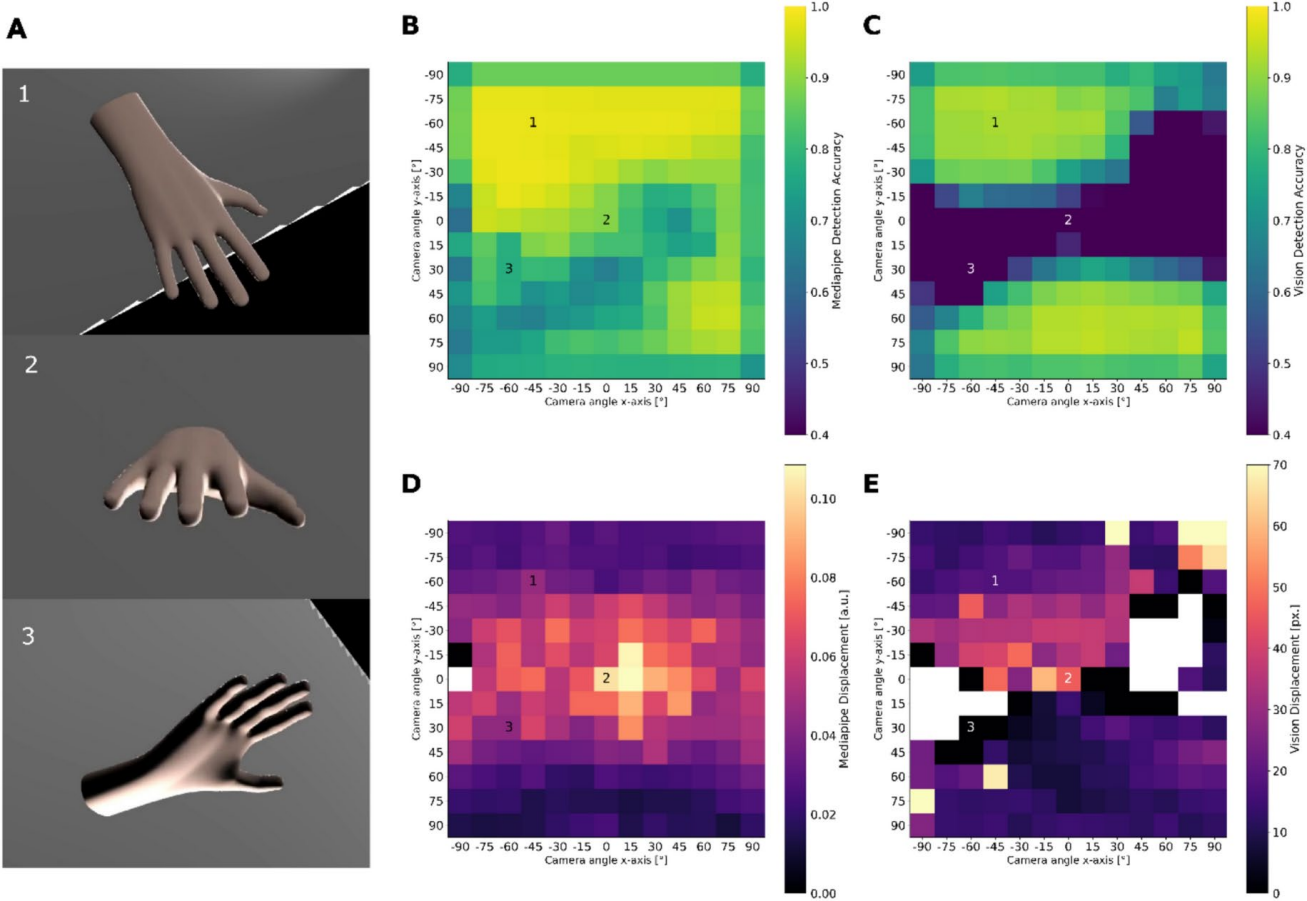
The upper heatmaps illustrate the estimated accuracy of hand detection on the simulated videos for Mediapipe (MP) (**B**) and Vision (VI) (**C**). The angle of the camera relative to the hand was systematically manipulated using “Blender”^23^. The rendered images give an idea of how the angle pairs represent the camera viewpoint (**A**). 1 shows the camera viewpoint at 0/0 (x-axis/y-axis), 2 the viewpoint at − 45/− 60, and 3 the viewpoint at 30/− 60. From this result, we concluded that a slightly elevated camera position is most likely to provide stable hand detection. However, the lower heatmaps illustrating the estimated logarithm of the tremor amplitude using the x/y/z coordinates of the MP norm (**D**) and VI using the x/y axis (**E**) suggest that there is a trade-off between accuracy and estimated amplitude depending on the camera viewpoint relative to the hand. For amplitude estimation using only the MP norm x and y data, see Suppl. Figure 3. The minimum color map value was set to 0.4 in (**B**, **C**) and the maximum color map value was set to 70 in (**E**) due to strong outliers.

To understand the effect of camera angle on frame accuracy and amplitude, we simulated a steady hand tremor. This simulated hand tremor, generated in a 3D simulation software, provides ground truth data for frequency and amplitude. With this approach, the CV algorithms MP and VI can be evaluated on the ground truth tremor motion only. Both MP and VI provide an internal measure of the likely accuracy of their hand detection models, and we used the models’ landmark position outputs to evaluate the amplitude. In a first simulation, the camera angle was rotated around the virtual hand in steps of 15 degrees, essentially covering a half-sphere of possible camera positions relative to the hand (Fig. 1A). The simulation showed better accuracy when the hand was viewed from above and preferably slightly to the side (Fig. 1). The more the camera view changed to a frontal (orthogonal) view, the more the accuracy decreased. This phenomenon was more pronounced for VI (Fig. 1B, C). Amplitude estimates using the MP norm (with and without z-axis) and VI showed a roughly inverted pattern. The estimated amplitude was higher when the camera angle was more orthogonal to the hand position (Fig. 1D, E). Logically, for views with very low accuracy, no amplitude could be estimated due to too many undetected frames (Fig. 1E).

**Fig. 2:**
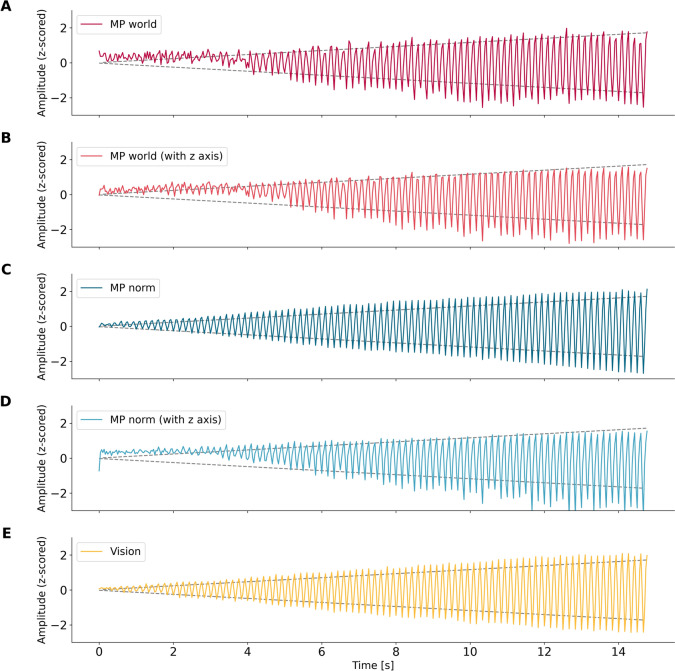
The plots show the z-score normalized amplitude estimates of the CV framework over time. The dotted line in the background depicts the total amplitude increase of the simulated hand as a reference. The different plots show the traces of different CV algorithms. They differ in how they detect hand landmarks in frame-by-frame videos. MP world = Mediapipe world landmarks (x/y), MP world z-axis = Mediapipe world landmarks (x/y/z), MP norm = Mediapipe normalized landmarks (x/y), MP norm (z-axis) = Mediapipe normalized landmarks (x/y/z), VI = Vision (x/y).

In a second simulation, we evaluated the performance of the CV framework in estimating the amplitude of the movement. We simulated a shaking hand with a linearly increasing amplitude. As shown in Fig. 2, the MP estimates of the world landmarks could only roughly follow the change in amplitude. Regarding the normalized MP estimates, it is clear that including the z-axis (Fig. 2B, D) has a negative effect on the estimation accuracy compared to using only the y- and x-axes (Fig. 2A, C, E), in the latter case the amplitude change is modeled more accurately. Using the same simulation, we investigated whether the detection of the peak frequency depends on the amplitude of the hand. We found that the peak frequency at 6 Hz, which was the ground truth, was robustly estimated throughout the simulation. While the frequency could still be determined for very small amplitudes, using the MP norm including the z-axis, MP world, and MP world with z-axis led to noisier frequency estimates (Supplementary Fig. 4).

### Validation on patient data

Data from a total of 20 tremor patients with different aetiologies are presented below (see Methods for details). Recordings of their hands were made using IMU, OMC, and video. The video camera was positioned according to the best detection accuracy determined in the virtual experiment.

### Amplitude estimation

**Fig. 3:**
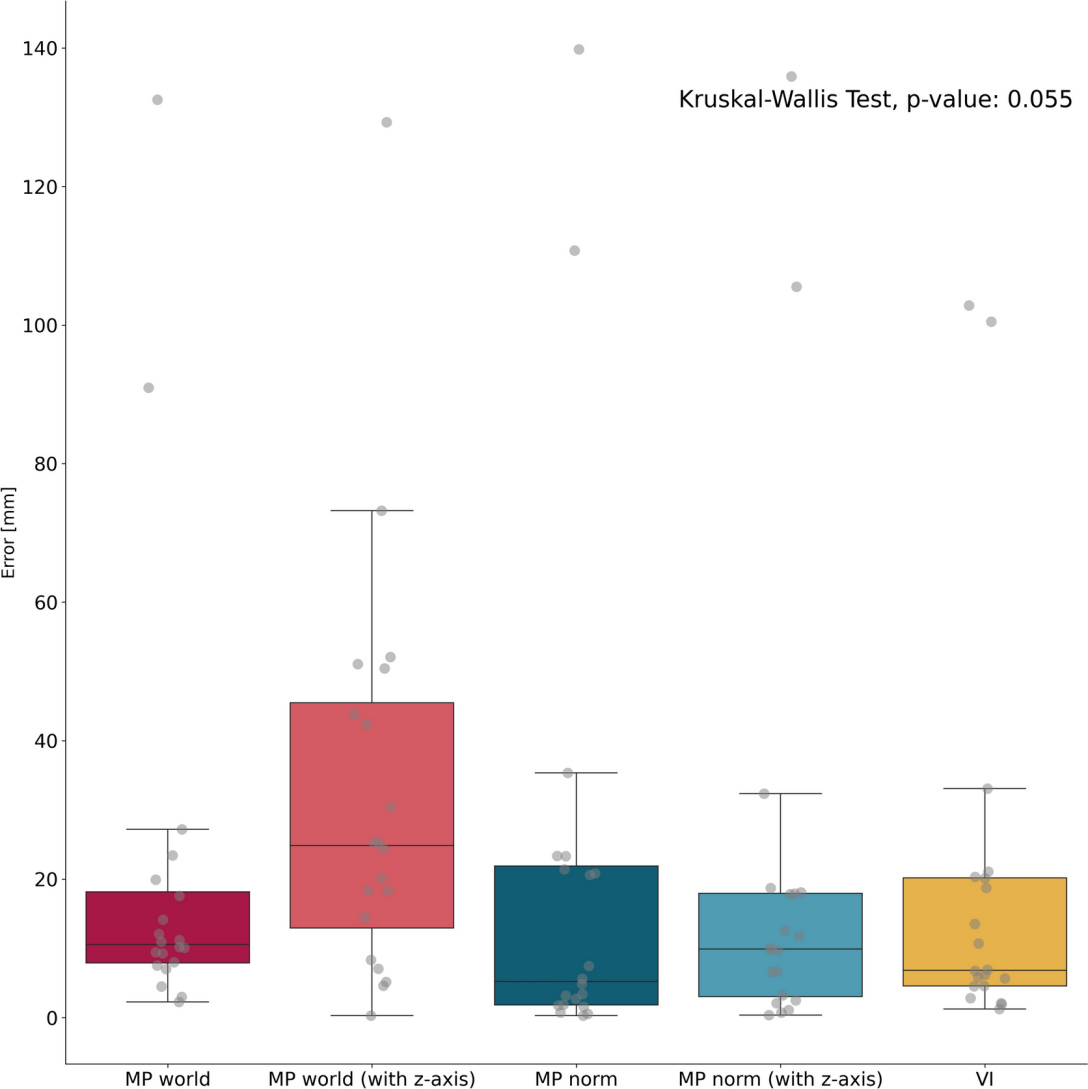
Boxplots showing the absolute difference of the median tremor amplitude between OMC and the CV frameworks. The differences in amplitude estimation were not significant. MP world = Mediapipe world landmarks (x/y), MP world z-axis = Mediapipe world landmarks (x/y/z), MP norm = Mediapipe normalized landmarks (x/y), MP norm (z-axis) = Mediapipe normalized landmarks (x/y/z), VI = Vision (x/y).

No significant differences were found when comparing the absolute estimation error of the tremor amplitude measured by CV with the reference OMC amplitude measurement. The median estimation error in millimeters was lowest for MP norm with 5 mm (MP world: 11 mm, MP world z-axis: 25 mm, MP norm z axis: 10 mm, VI: 7 mm, see Fig. 3, suppl. Table 1, suppl. Figure 5). The differences were not statistically significant.

**Fig. 4:**
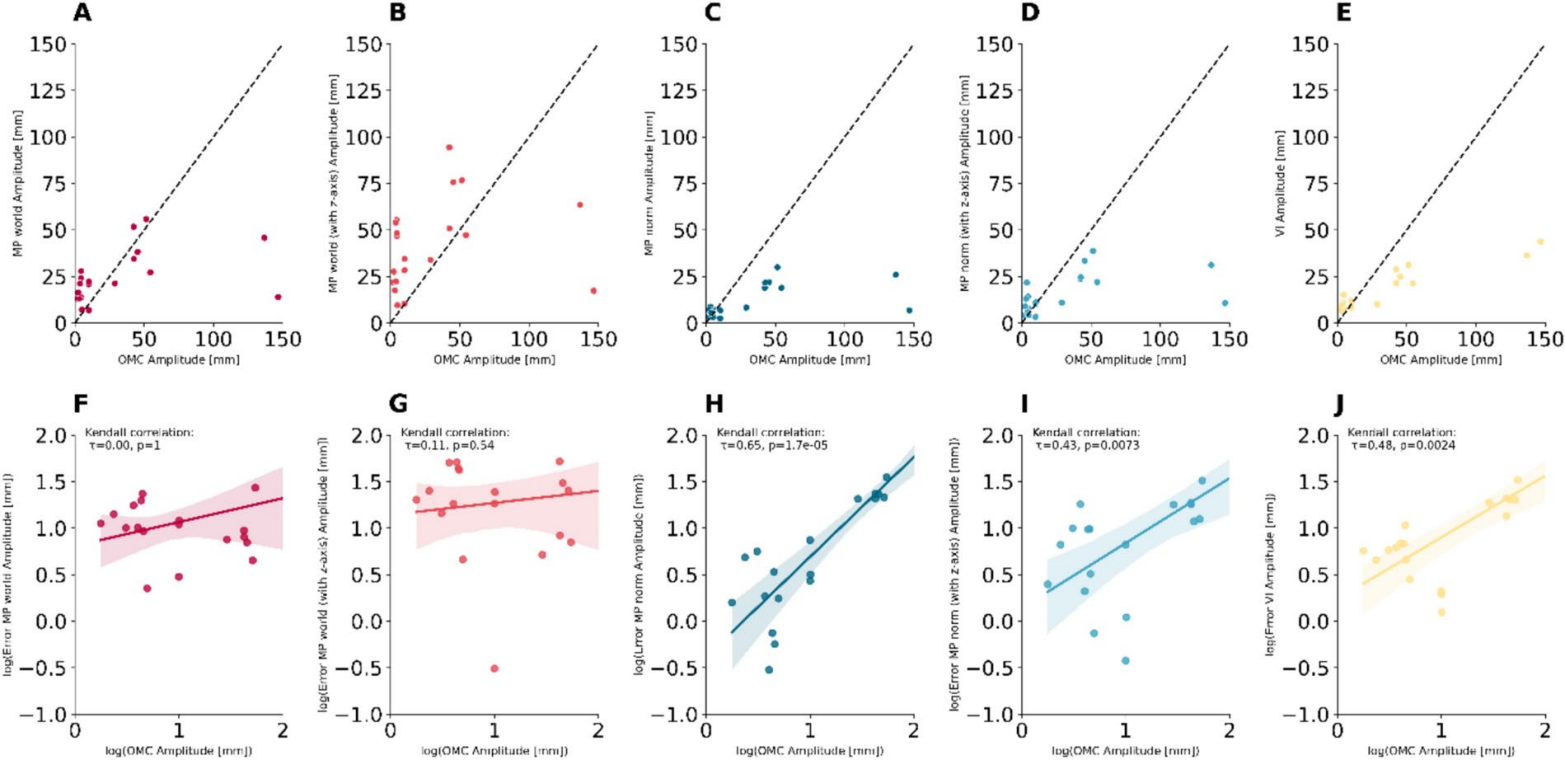
The scatterplots (**A**–**E**) depict the amplitude estimation results of the CV frameworks versus the ground OMC considered as ground-truth. The subplots (**F**–**J**) show the dependencies of the OMC amplitude as ground-truth and the estimation error of the CV methods. Significant correlations revealed systematic errors in amplitude estimation for MP norm, MP norm with z-axis and VI. For better visibility the OMC amplitude on the x-axis and the amplitude estimation error of the different methods were log-transformed. Kendall’s correlation is a rank-based method which is independent of log-transform and less sensitive to outliers. OMC = Optical Motion Capture, MP world = Mediapipe world landmarks (x/y), MP norm = Mediapipe normalized landmarks (x/y), VI = Vision (x/y).

We observed significant correlations of the ground-truth OMC amplitude and the estimation error for MP norm (t = 0.65, *p* =  < 0.001), MP norm z axis (t = 0.43, *p* =  < 0.0073) and VI (t = 0.48, *p* =  < 0.0024) suggesting a systematic error. These correlations were not found for MP world and MP world z-axis (Fig. 4).

**Fig. 5:**
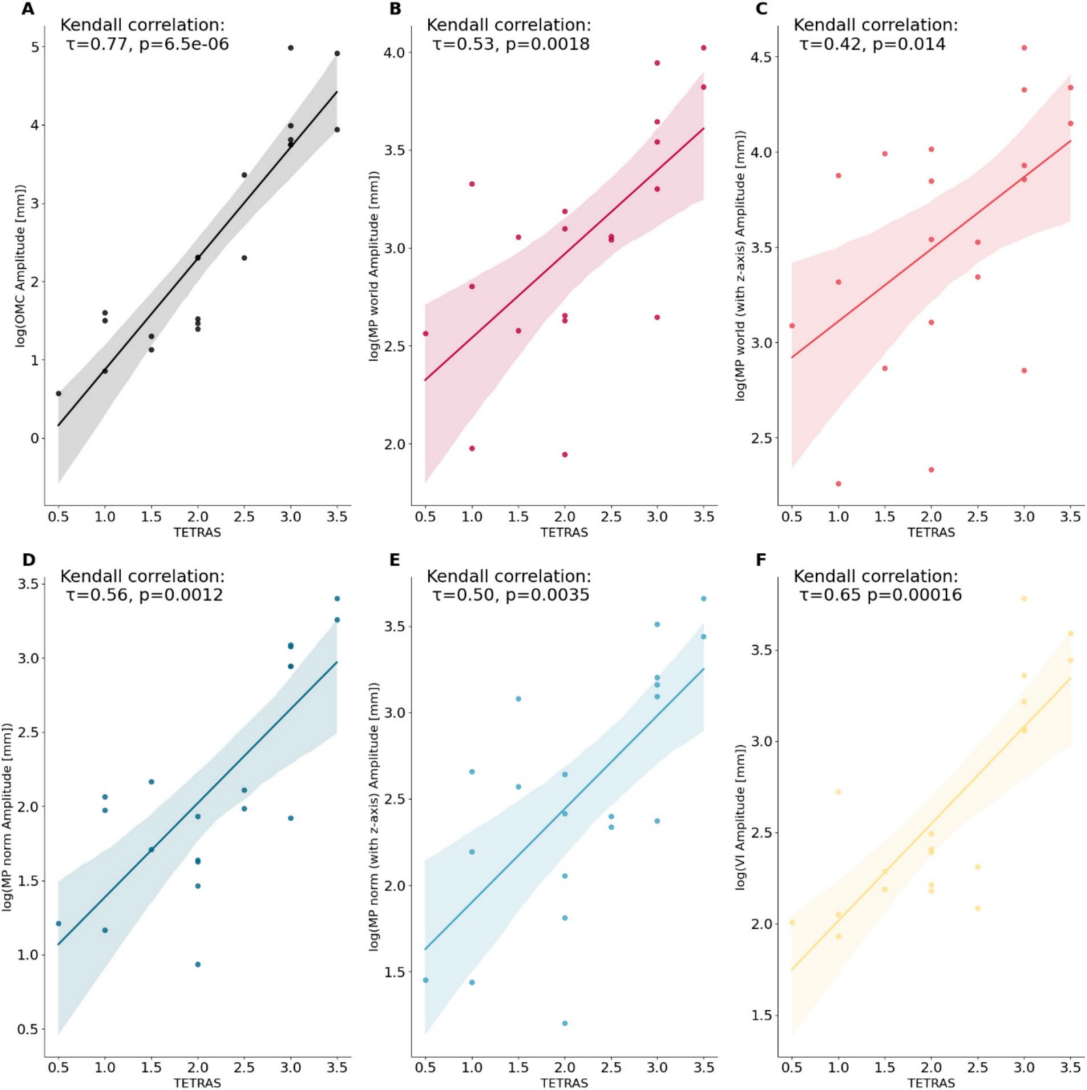
Plots showing the correlations between the log-transformed amplitude estimations of all methods (**A**: OMC,** B**: MP world,** C**: MP world z-axis,** D**: MP norm,** E**: MP norm z-axis,** F**: VI) and the Essential Tremor Rating Assessment Scale ratings (TETRAS) of postural or resting tremor. Except for log-transformed amplitude estimates, all methods showed a significant correlation with clinical TETRAS scores. MP world = Mediapipe world landmarks (x/y), MP norm = Mediapipe normalized landmarks (x/y), VI = Vision (x/y).

All frameworks showed a significant correlation with the clinical TETRAS scores (Fig. 5). However, Fig. 5 also shows that the estimated log-transformations of amplitudes associated with different TETRAS scores often overlap with the preceding and subsequent scores. This is even true for the log transformation of the amplitude measured with OMC, which correlates most strongly with the clinician’s TETRAS ratings^20^.

### Peak frequency estimation

**Fig. 6:**
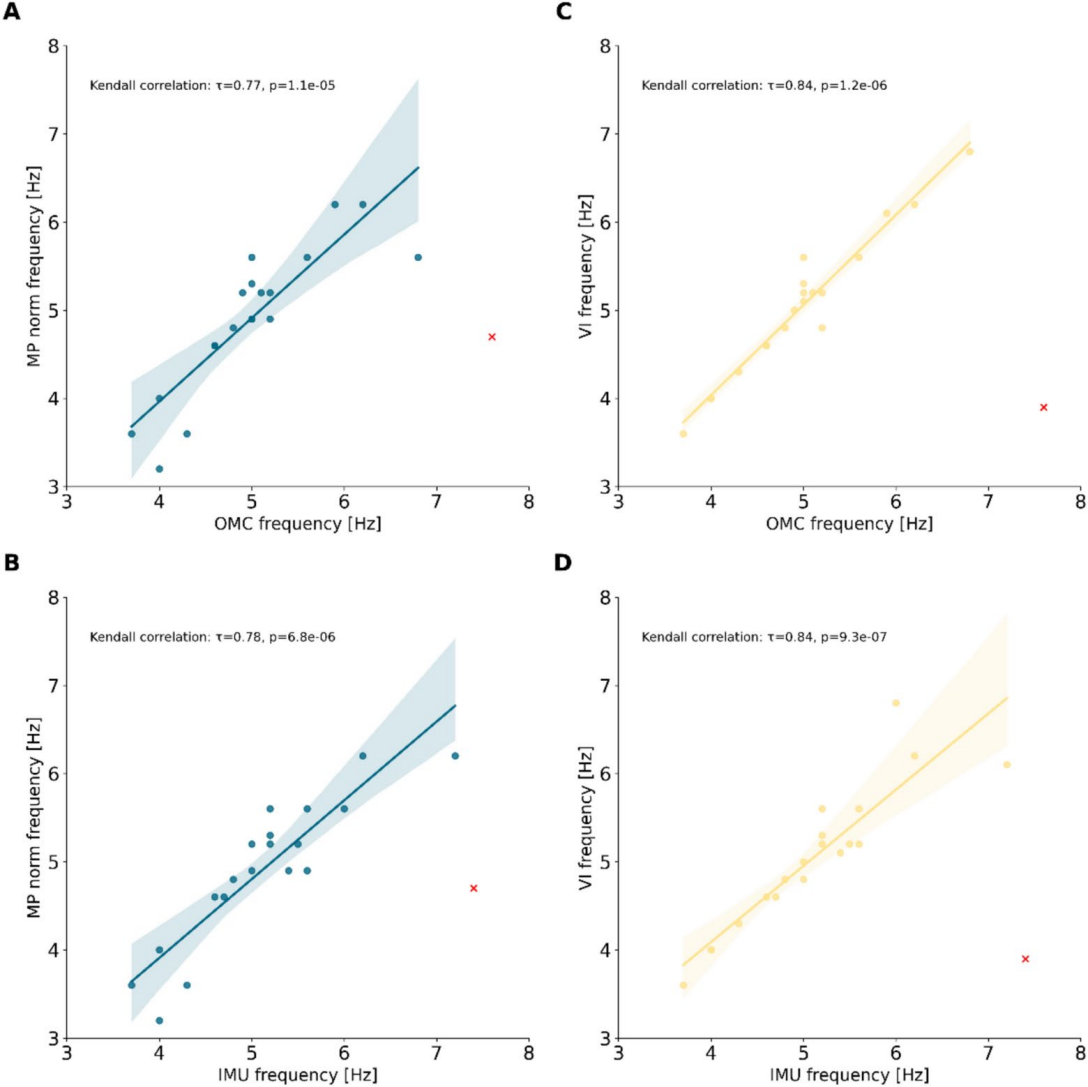
These plots show the correlations between estimated peak frequency in Hz between (**A**) MP norm and OMC, (**B**) VI and OMC, (**C**) MP norm and IMU and (**D**) VI and IMU. Peak frequency estimation did not succeed for subject 8 which had the lowest tremor amplitude of all subject. (marked with red cross). OMC = Optical Motion Capture, IMU = Inertial Measurement Unit, MP norm = Mediapipe normalized landmarks (x/y).

As shown in the simulated experiment, the estimation of a z-axis for MP norm and MP world introduces noise into the estimation of the tremor peak frequency (Fig. 2, suppl. Figure 4). Therefore, we examined the detected peak frequencies of MP norm and VI and compared them with the peak frequencies derived from OMC and IMU recordings (Fig. 6, suppl. Figure 6). Both the MP norm and VI peak frequency estimation resulted in comparably small median estimation errors (MP norm vs OMC: 0.1 Hz, MP norm vs IMU 0.15 Hz, VI vs OMC 0.05 Hz, VI vs IMU 0.1 Hz, for Minimum, Maximum and IQR^25–75^ see suppl. Table 3). The peak frequency of the subject with the lowest tremor amplitude (TETRAS 0.5) could not be estimated (Fig. 6). An absolute error of peak frequency estimation of greater than 1 Hz was considered insufficient accuracy (Fig. 6, red crosses).

## Discussion

In this study we investigated the ability of CV frameworks to measure characteristics of hand tremor in simulated and real data. In a first step, we evaluated whether the estimation accuracy and movement amplitude depend on positioning of the hand and camera relative to each other, using a synthetic data approach. In a second step, we collected a video data set of 20 patients with hand tremor, synchronized with OMC and IMU recordings. We compared the tremor amplitudes and frequencies estimated by the two CV frameworks (MP and VI). We found that both provided reliable measures of tremor peak frequency (Fig. 6). However, the accuracy of the q-amplitude estimation depended significantly on the configuration of the framework. Finally, we found significant correlations between the CV framework amplitude estimates and the TETRAS scores, demonstrating the clinically relevant capabilities of such approaches (Fig. 5). However, the tremor amplitude estimates were less accurate compared to the gold standard and showed a dependence on the actual magnitude of the tremor (Fig. 4).

### Amplitude estimation

In summary, we found the amplitude estimates to be less reliable than the frequency estimates for several reasons. At first glance, the median estimation error of e.g. the MP norm is as small as 5 mm, but we found that the estimation residuals depend systematically on the ground-truth OMC amplitude (Suppl. Table 1, Fig. 4). While this systematic influence could not be statistically confirmed for MP world and MP world with z-axis, Fig. 4 shows how far the estimated amplitude for the two subjects with larger tremor amplitudes deviates from the ground truth in all CV frames examined. With a relatively small group size, a systemic error cannot be excluded for MP world and MP world with z-axis.

Second, the perceived and clinical severity of tremor does not increase linearly. Instead, it follows a logarithmic pattern, as described by the Weber-Fechner law^26^. This means that the clinical perception of differences in tremor amplitude depends more on the relative magnitude rather than the absolute magnitude. Therefore, relative estimation is important in assessing clinical applicability. Unfortunately, this is complicated by the phenomenon that the relative error increases exponentially as the ground truth scale converges to the estimation accuracy of a method (Suppl. Figure 5). With this in mind, we also interpreted the amplitude estimation errors relative to the corresponding OMC amplitude (Supplementary Table 2, Suppl. Figure 5). For example, the MP world, which at least in our cohort had no statistically significant system error, had a median relative error of 100%. In a clinical setting, this would be a scale that could potentially lead to severe over- or underestimation of tremor severity. In this context, the significant correlations of the CV framework amplitude estimate with the TETRAS ratings should not be misinterpreted as proof of accurate CV-based tremor amplitude estimation. As shown in Fig. 5, the variability and class overlap can be pronounced. In addition, the clinical TETRAS ratings and other scores are themselves only estimates of tremor amplitude and should not be considered a gold standard. Notably, the logarithmic OMC amplitude correlated highly with the TETRAS ratings, but could not perfectly discriminate the TETRAS rating classes.

Nevertheless, under standardized assessment conditions, our results support the internal validity of the CV methods, suggesting that coarse qualitative differences in the amplitude of a tremor can indeed be detected. This is further supported by the simulation results, which show that the estimated amplitude dimensions are consistent with the increase in simulated amplitude (Fig. 2). Clinically, this means that within a recording of a tremor, changes in amplitude can be detected, which is helpful, for example, in the diagnosis of functional tremor^12^.

In conclusion, intra-individual and inter-session comparisons using CV for tremor analysis should be interpreted with caution. To understand the inconsistent performance of amplitude estimation in our data, one has to consider that on the one hand the estimated tremor amplitude depends on the camera position relative to the patient’s hand, but on the other hand the accuracy of hand detection also depends on the camera position (Fig. 1). Faced with this trade-off, we chose a camera angle that provided greater tracking accuracy in our real-world data. MP’s estimation of the 3rd dimension was not accurate enough to compensate for the influence of camera perspective and essentially introduced more noise into our measurements. The developers of MP themselves state that it can be considered a 2.5D approach, since the z-axis represents an estimate of depth^14^. While more sophisticated optical calibration techniques could potentially have improved amplitude estimation, we argue that the need for time-consuming optical calibration would contradict the ideal application of simply pulling out a smartphone and performing a tremor analysis.

### Frequency estimation

While amplitude determines the clinical burden of tremor from the patient’s perspective, analysis of tremor frequency is of interest for diagnosis and research. Our results show that accurate peak frequency detection can be achieved using both the MP and VI frameworks with a median error as small as 0.05–0.15 Hz depending on the reference (OMC or IMU). Consistent with the failure of peak frequency estimation for the subject with the mildest tremor (Fig. 6), the accuracy of peak frequency estimation showed significant negative correlations with actual tremor amplitude (Suppl. Figure 7). However, the size of our data set is not sufficient to determine the minimum amplitude required for frequency peak detection.

### Comparison with previous works

Pintea et al. compared two frameworks for CV-based tremor analysis with synchronized accelerometry-based analysis. 55 patients were assessed in a variety of tasks, with the best approach resulting in a mean average error of approximately 1 Hz relative to the accelerometry-based ground truth^8^. Williams et al. evaluated an optical flow-based approach on videos of a total of 37 patients with various hand tremor entities. The results were validated against synchronized uniaxial accelerometry. While the proof of principle of accurate tremor frequency estimation from videos was achieved, amplitude estimation was not explicitly investigated^10^. Consistent with our findings, the frequency estimation error was not related to amplitude, which in this case was assessed with clinical ratings.

Park et al. analyzed videos of Unified Parkinsons Disease Rating Scale (UPDRS) Part III examinations of Parkinson patients with aid of the 2D pose estimation framework “OpenPose”^21^.This information was then fed into a support vector machine to automatically score rest tremor and bradykinesia. This resulted in good overlap between machine and human UPDRS III rest tremor ratings^22^.

Similarly, Fois et al. applied the same framework to videos of rest tremor and finger-to-nose tremor examinations. Neurophysiological measures (IMU, EMG) were available in this cohort. The estimated logarithmic peak powers of this approach showed a convincing correlation with clinical ratings. However, for peak frequencies, the overlap between the CV-based method and the neurophysiological recordings was poor^9^.

Another recent work by Wang et al. applied the MP hand detection framework to the dataset originally recorded by Pintea et al. to detect hand tremor by feeding the retrieved information into various classification algorithms to determine whether hand tremor was present. Information on more specific characteristics of the tremors was not reported^11^.

A study using MP by Güney et al. examined postural tremor in PD patients, comparing MP with accelerometry. Again, the use of CV for frequency estimation was confirmed. However, the reported perfect correlation between amplitude estimation using MP and accelerometry was invalidated by one outlier^13^.

Recently, Friedrich et al. compared the Mediapipe framework (using the estimated x, y, and z coordinates) to OMC as ground truth, examining postural and kinetic tremor in 8 patients^3^. To circumvent the poor detection accuracy of MP in a frontal view relative to the hand (see Fig. 1), a custom 2D handtracking model was trained. The authors report excellent results for frequency estimation and good results for amplitude estimation in their cohort. While our analysis found the lowest median estimation error of 7 mm for the VI frame, there is a systematic estimation error for low tremor amplitudes when expressed relative to ground truth (Fig. 4). Furthermore, in a larger retrospective cohort, significant correlations were found between TETRAS ratings of tremor severity and sensitivity and changes in tremor amplitude after DBS. The estimated amplitude was derived from the power of the tremor peak in the frequency spectrum^3^.

In summary, previous works have used optical flow, open-pose body pose tracking, and MP hand tracking to assess tremor in videos. While tremor frequency estimation based on CV is relatively accurate in different works, correct amplitude estimation remains a challenge. We conclude that significant correlations between clinical ratings of tremor and amplitude estimation have been found across studies^3,9,22^. Whether the CV for tremor analysis in its current state is accurate enough for clinical application needs to be critically assessed in larger cohorts that take into account the technical limitation we systematically examined.

## Conclusions

Our results indicate that both the MediaPipe and Vision frameworks provide accurate estimates of tremor peak frequency and a baseline estimate of tremor amplitude, although neither CV framework was specifically designed for tremor analysis. However, further validation and improvements in CV-based tremor analysis are essential before its widespread clinical application, particularly in the accurate estimation of tremor amplitudes. With some confidence, we can expect that rapid advances in computer vision technology will significantly improve hand pose estimation, thereby improving the quality of noncontact tremor analysis.

## Methods

### Simulated data

**Fig. 7:**
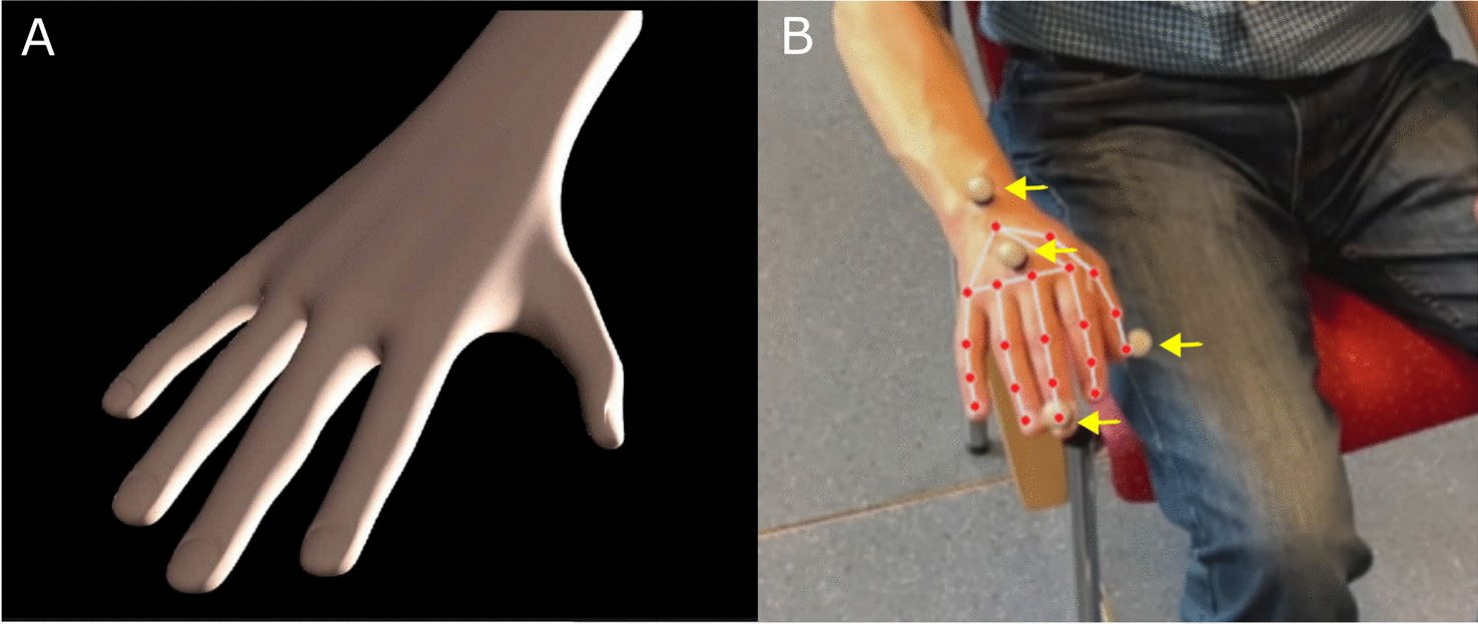
Experimental Setup. The left side (**A**) shows the simulated hand in blender. Important tremor features could be manipulated. On the right side (**B**) the real-world measurement setup is shown. Optical motion capture markers (yellow arrows) were attached to the wrist, the base and the tip of the middle finger and the tip of the thump. The green box depicts the position of the IMU attached to the palm. The red points and their connections are the 21 hand landmarks that are tracked by the Mediapipe (MP) and Vision (VI) framework (see also suppl. Figure 1).

To obtain the generated data, a 3D hand model was simulated in Blender to shake under fully controlled conditions^23^. We simulated hand movements in Blender and rendered videos from Blender to pass to MP. The amplitude of the hand movement, the tremor frequency and the angle of the camera to the hand were manipulated. Both frameworks were able to recognize the simulated hand (Fig. 7, suppl. Figure 2).

### Acquisition of real-world data

#### Clinical data

Twenty patients with tremor were consecutively recruited from the tremor outpatient clinic of the Department of Neurology at the University Hospital of Kiel, Germany, and gave their written informed consent to participate in the study and to have exemplary figures of their hands published in a scientific publication. The study was conducted in accordance with the Declaration of Helsinki and other relevant guidelines and regulations. The ethics committee of the University of Kiel approved the study protocol. Tremor was assessed by an experienced movement disorder specialist using The Essential Tremor Rating Assessment Scale (TETRAS) (Table 1)^24^. Since the TETRAS does not encompass a rest tremor item, we applied the postural tremor item of the TETRAS for assessing rest tremor using the same limits (suppl. Table 4). This approach has been chosen by others before^25^. Real-world videos of tremor patients were recorded using a smartphone camera in slow-motion capture mode (Apple iPhone 12 mini, 120 Hz, HD resolution 1920 × 1080 pixels), a 12-camera optical motion capture system (Qualysis, 340 Hz/100 Hz), and IMUs (Noraxon myoMotion, 200 Hz). In cases of bilateral tremor, the more severely affected hand was examined. Motion capture markers were attached to anatomical landmarks. (see suppl. Figure 1 + 2). The IMU was taped to the palm of the hand to avoid interference with the Mediapipe algorithm. Patients were seated in a chair for 30-s recordings. They were instructed to pose the arms in the position in which the tremor occurred (resting or postural tremor). The camera was mounted on a tripod at a distance of 1.5 m from the chair. The camera view was focused on the hands from an upper frontal position (Fig. 7). This position was chosen based on the results of the virtual part of this study. The derived data and videos were stored locally and further analyzed in an offline pipeline. All patients (expect the functional and physiological tremor cases) were on their regular medication.

**Table 1:** Clinical characteristics of the examined tremor patients.

Disease duration (years)	10.5 (5.9)
Tremor type (rest/postural)	7/13
TETRAS Rating of the measured upper extremity (0–5)*	2.175 (0.89)
Diagnosis	
ET	8
ET+**	5
PD	5
Functional tremor	1
Physiological tremor	1

*Rating of postural or resting tremor amplitude.

**questionable dystonic posturing in 4/5 and resting tremor in 1/5 cases.

ET = Essential Tremor, ET+  = Essential Tremor plus, PD = Parkinson’s Disease.

### Analysis pipeline

**Fig. 8:**
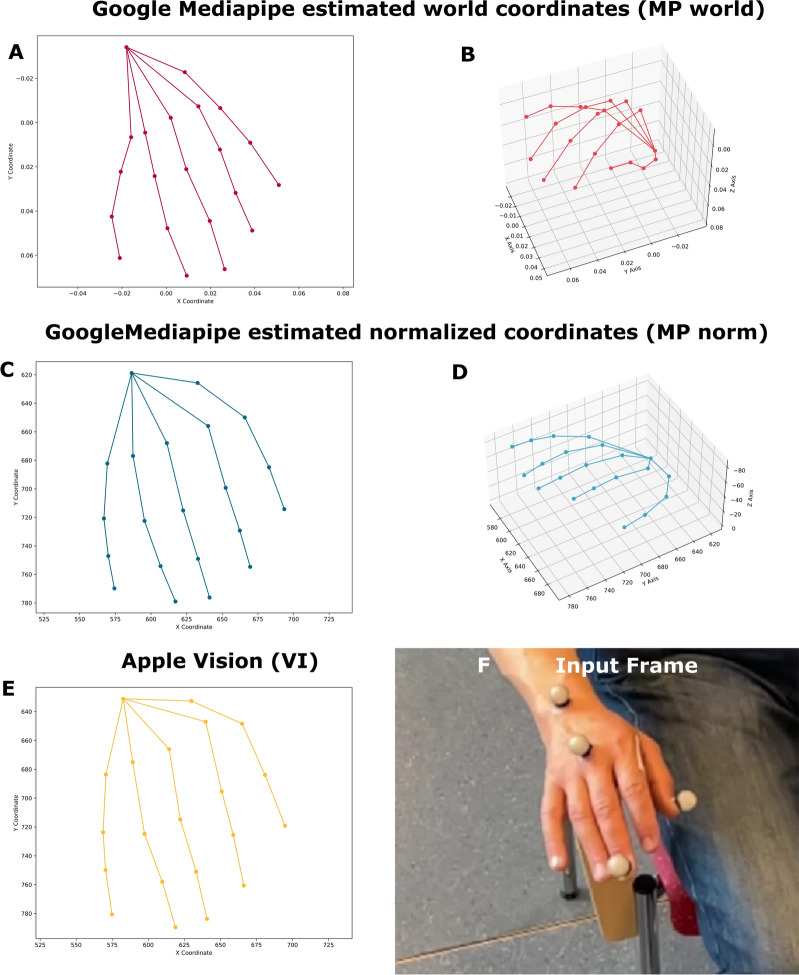
CV algorithms and processing. These plots illustrate qualitative differences between all five examined methods. MP world = Mediapipe world landmarks (x/y), MP norm = Mediapipe normalized landmarks (x/y), VI = Vision (x/y).

After data acquisition, the MP and VI hand tracking algorithms were applied to both the real world and the simulated videos. MP returns the estimated x-, y- and z-positions of 21 tracked points relative to the width and height of the video frame. The z-axis is described as having roughly the same scale as the x-axis^14^. VI returns the x- and y-positions of 21 landmarks at the same locations as MP (Fig. 8, suppl. Figure 1). The positions are returned in x and y-pixel positions^15^. For optical motion capture, we tracked the x, y, and z positions of the tip of the thumb, the metacarpophalangeal joint of the middle finger, and the tip of the middle finger. These points correspond to points 4, 9, and 12 of the MP hand tracking (Fig. 7). For further evaluation, only point 12 (middle finger tip) was examined. The IMU stored the x, y, and z acceleration data. A band-pass filter of 2–10 Hz was then applied. The lower limit of the bandpass filter was used to ensure the absence of voluntary motion artifacts or jitter due to tracking inaccuracies in the signal.

### Amplitude estimation

**Fig. 9:**
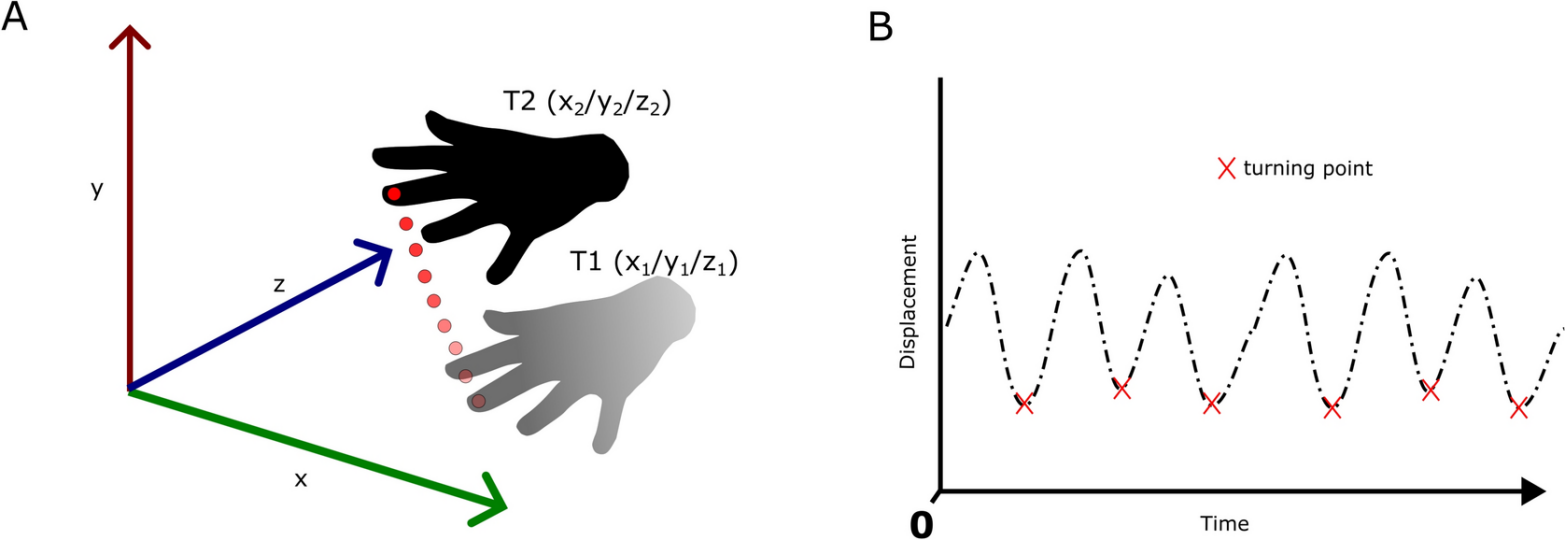
This figure illustrates the amplitude estimation algorithm. The Euclidean distance between each successively tracked middle fingertip was calculated. The resulting displacement signal was band-pass filtered between 2–10 Hz. The time point with the smallest displacement between two consecutive points was detected as the turning point during the shaking movement. The sum of the displacement of the unfiltered signal between two consecutive inflection points was calculated as the amplitude of a hand swing. The median of these amplitudes was calculated to estimate the total tremor amplitude.

For amplitude estimation, the distance between each consecutive point of the x/y/(z) locations measured by MP, VI, and OMC of the tip of the middle finger was calculated. To calculate approximate scaling factors, the pixel size of the 9.5 mm OMC markers was measured in the x and y dimensions. Since the wrist was fixed to the chair back, we assumed that the main amount of movement would be roughly in an x/y plane (Fig. 7). The camera perspective was not taken into account as all videos were recorded under the same conditions. MP returns both normalized values and estimated “world coordinate landmarks”. No scaling factor was applied to the latter. The resulting distances of consecutive points over time were band-pass filtered between 2–10 Hz. Amplitudes were then extracted as shown in Fig. 9. Typically, clinicians judge the average tremor amplitude. To minimize the effect of potential outliers due to tracking error, we chose to compare the median amplitudes of MP, VI and OMC, all to each other and to the TETRAS ratings. For MP we distinguished between the following approaches, depending on which data were used for amplitude estimation: “MP world”: x/y world coordinates, “MP world z-axis”: x/y/z world coordinates, “MP norm”: x/y normalized coordinates, “MP norm z-axis”: x/y/z normalized coordinates. In case of the virtual experiment, we used the original output without scaling. For the analysis of real-world data, MP world measures were scaled from meters to millimeters, and MP standard measures and VI scaling factors were applied relative to the size of the OMC marker (9.5 mm diameter) attached to the tip of the middle finger. The OMC results were used as ground truth for evaluating amplitude estimation performance. The IMU data were not further evaluated in the context of amplitude estimation because positions are not directly measured, and the data would require double integration, which is prone to error and drift.

### Frequency peak detection

The x- and y-axis (plus z-axis for OMC and IMU) frequency spectra were estimated using Welch’s method. A frequency of interest (FOI) range of 3–8 Hz was defined in which peaks could be detected. Peak detection was performed on the derived spectra. The peaks with the highest power within the FOI were selected. The peak detection threshold was set at 10% of the maximum power of the spectrum. Based on prior knowledge from the simulation results, the frequency spectra were derived from the x- and y-axis of the MP only.

### Statistical analysis

Kendall’s tau was calculated for correlations between measures derived from real-world data. Group comparisons were made using the appropriate nonparametric or parametric test, depending on the distribution of the data.

## Supplementary Information

Supplementary Information.

## Data Availability

The code corresponding to our work, the anonymous handtracking data and ground truth data is available at https://github.com/JuliusWelzel/TremorComputerVision. The original videos and detailed clinical data cannot be shared due to legal restrictions.
