# Supplementary material for: Validity of tremor analysis using smartphone compatible computer vision frameworks
Source: Sci Rep. 2025 Apr 18;15:13391. doi: 10.1038/s41598-025-97252-4 (PMC12008214; doi:10.1038/s41598-025-97252-4)
Supplement: Supplementary file 1 — Supplementary Information. [file 41598_2025_97252_MOESM1_ESM.docx]

Validity of tremor analysis using smartphone compatible computer vision frameworks

Robin Wolke MD^1*+^, Julius Welzel MSc Psych^1*+^, Walter Maetzler MD^1^, Günther Deuschl MD^1^, Jos Becktepe MD^1^

*These authors contributed equally to this work.

^1^ Department of Neurology, UKSH, Kiel University, Kiel, Germany

^+^ Corresponding Authors: Robin Wolke, Julius Welzel

# Supplementary material

| 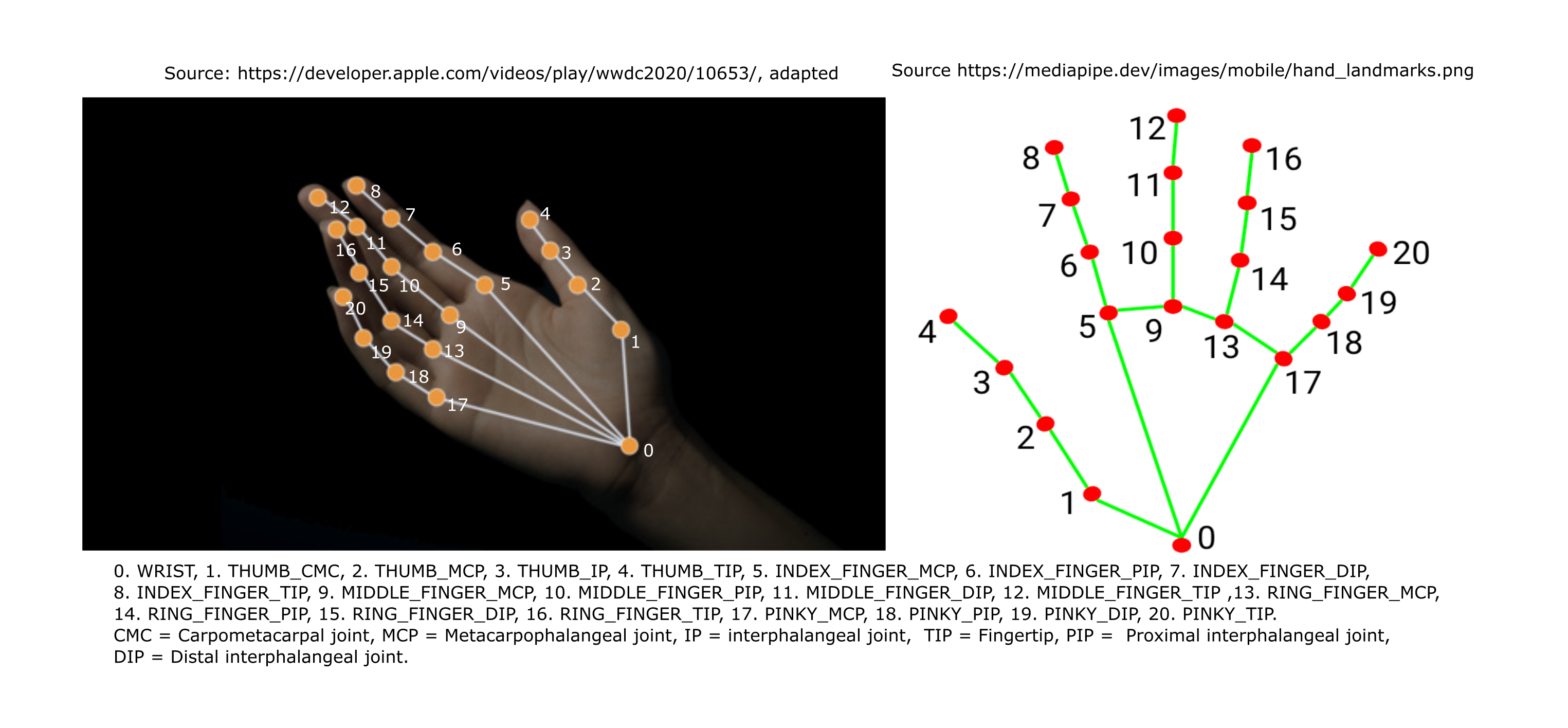  Supplementary Figure 1. This figure shows the 21 tracked points by VI (left) and MP (right). For both frameworks the tracked landmarks where the same. VI returned x/y positions in pixels related to the frames’ width and height, MP x/y/z positions normalized to the frames’ width and height as well as x/y/z positions in estimated world coordinates. |
| --- |

| 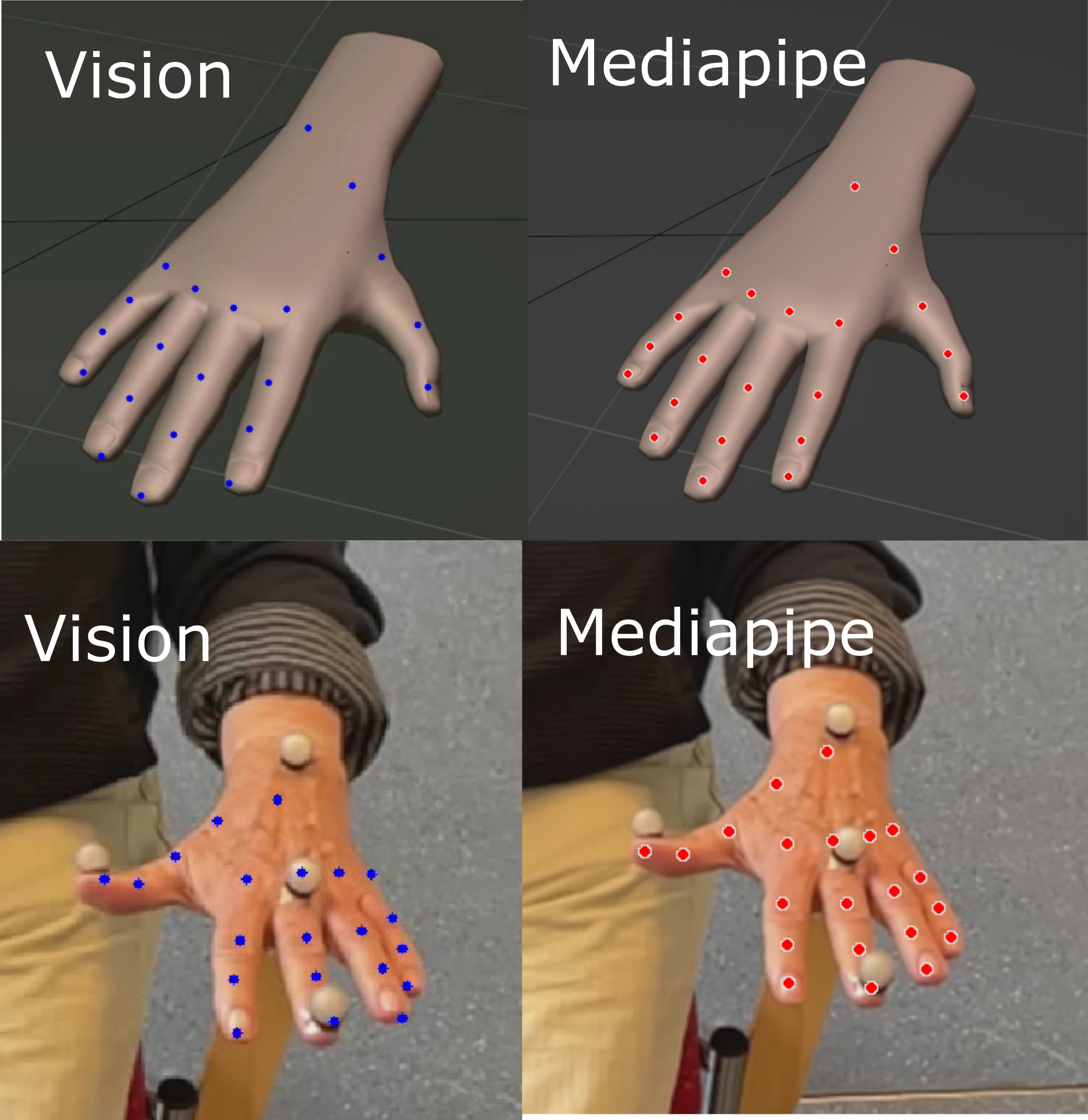  Supplementary Figure 2. Example of qualitative tracking results of VI and MP for simulated and real data. The OMC markers did not interfere severely with the tracking. |
| --- |

| 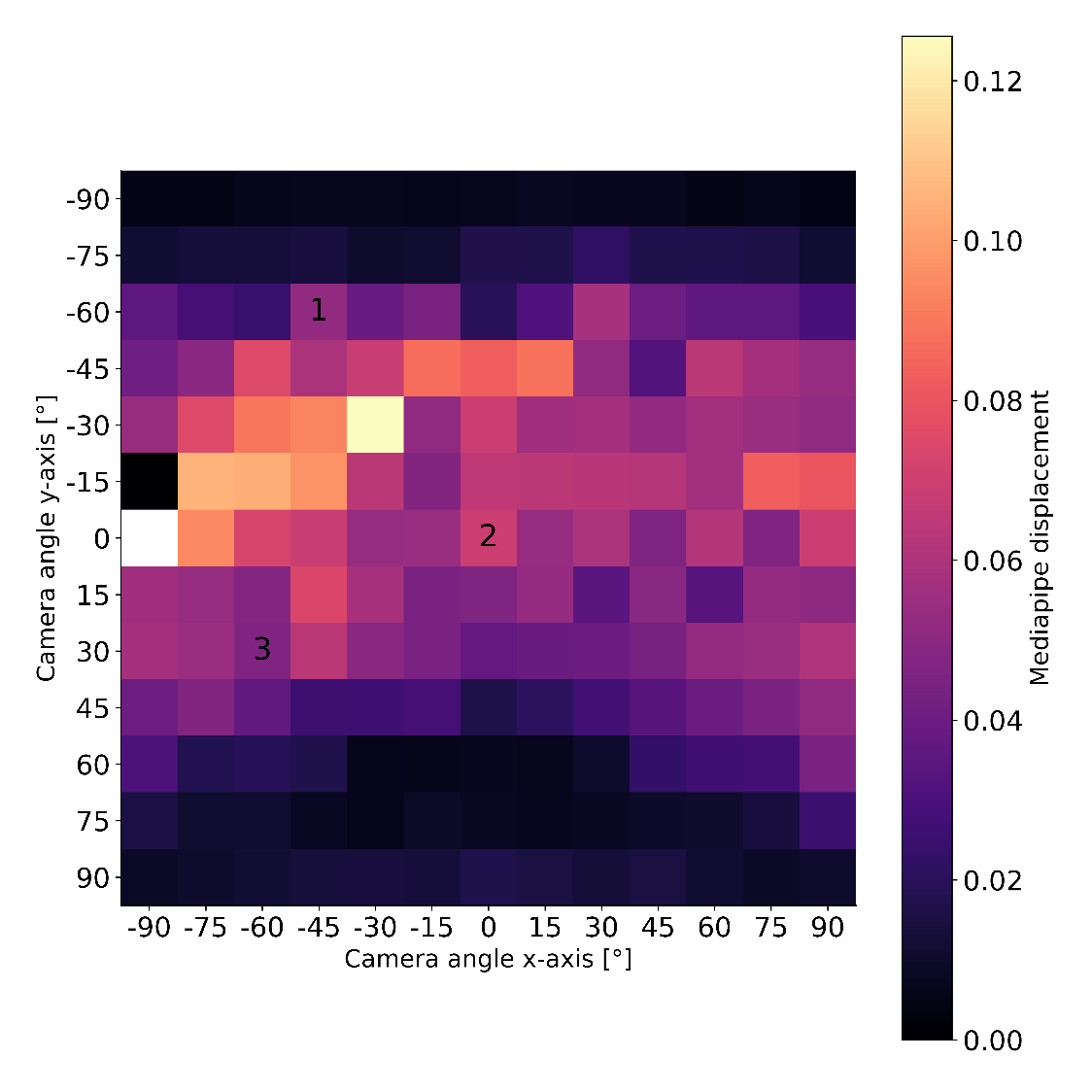  Supplementary Figure 3. Estimated amplitude using the x/y/z axis of MP norm depending on camera viewpoint. MP norm = Mediapipe normalized landmarks. |
| --- |

| 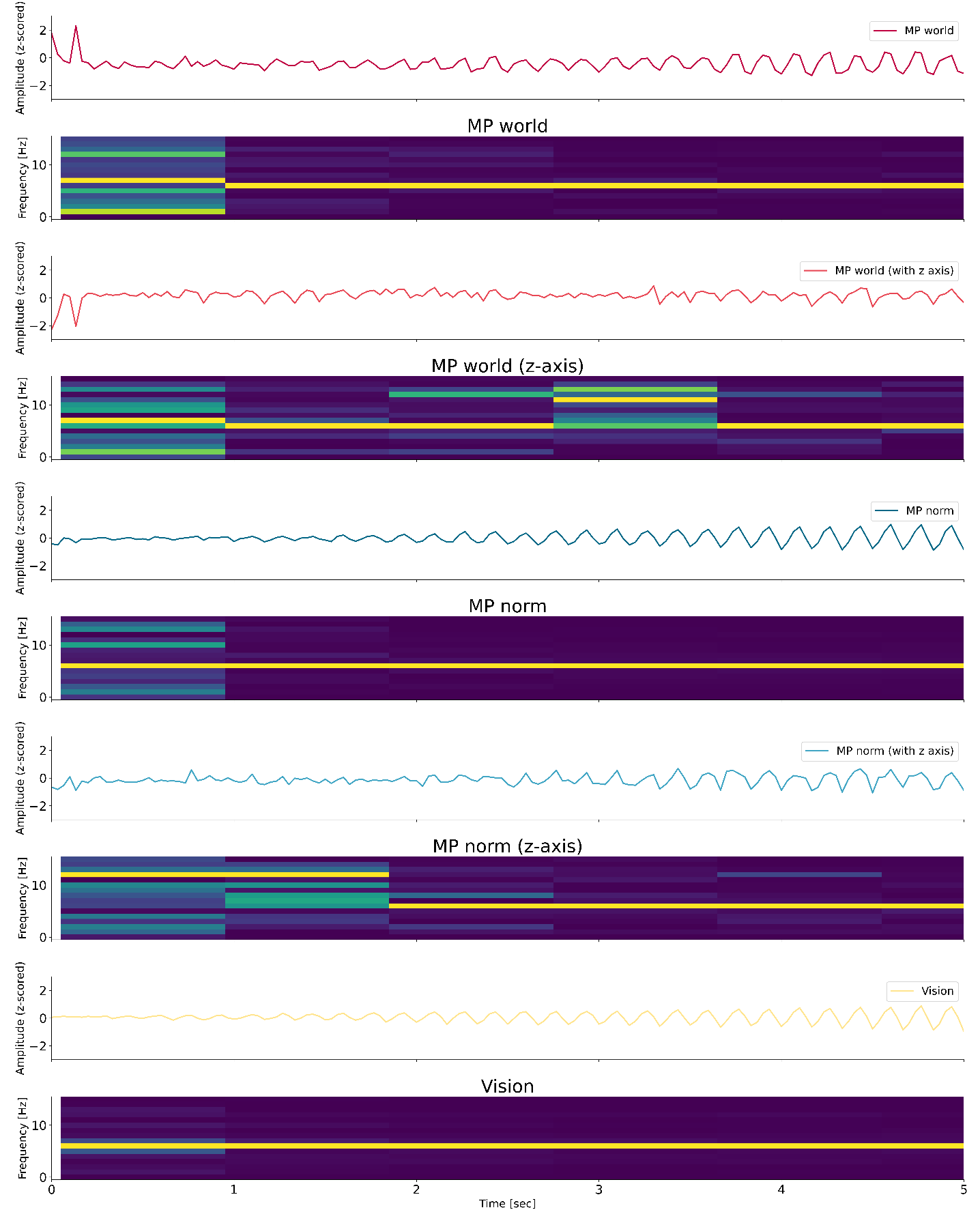  Supplementary Figure 4. First five seconds of the CV output and frequency spectra derived from the simulated hand with linearly increasing amplitude. The power of the frequency bins was normalized to the maximum power of each second separately. MP world, MP world with z axis and MP norm with z-axis show noisier frequency spectra when the tremor amplitudes are small. MP world = Mediapipe world landmarks, MP norm = Mediapipe normalized landmarks, VI = Vision (x/y). |
| --- |

Supplementary Table 1: Absolute differences to OMC in mm of the estimated median amplitude

|  | Median | Minimum | IQR 25 | IQR 75 | Maximum |
| --- | --- | --- | --- | --- | --- |
| MP world | 11 | 2 | 8 | 18 | 133 |
| MP world (z-axis) | 25 | 0 | 13 | 45 | 129 |
| MP norm | 5 | 0 | 2 | 22 | 140 |
| MP norm (z-axis) | 10 | 0 | 3 | 18 | 136 |
| VI | 7 | 1 | 5 | 20 | 103 |


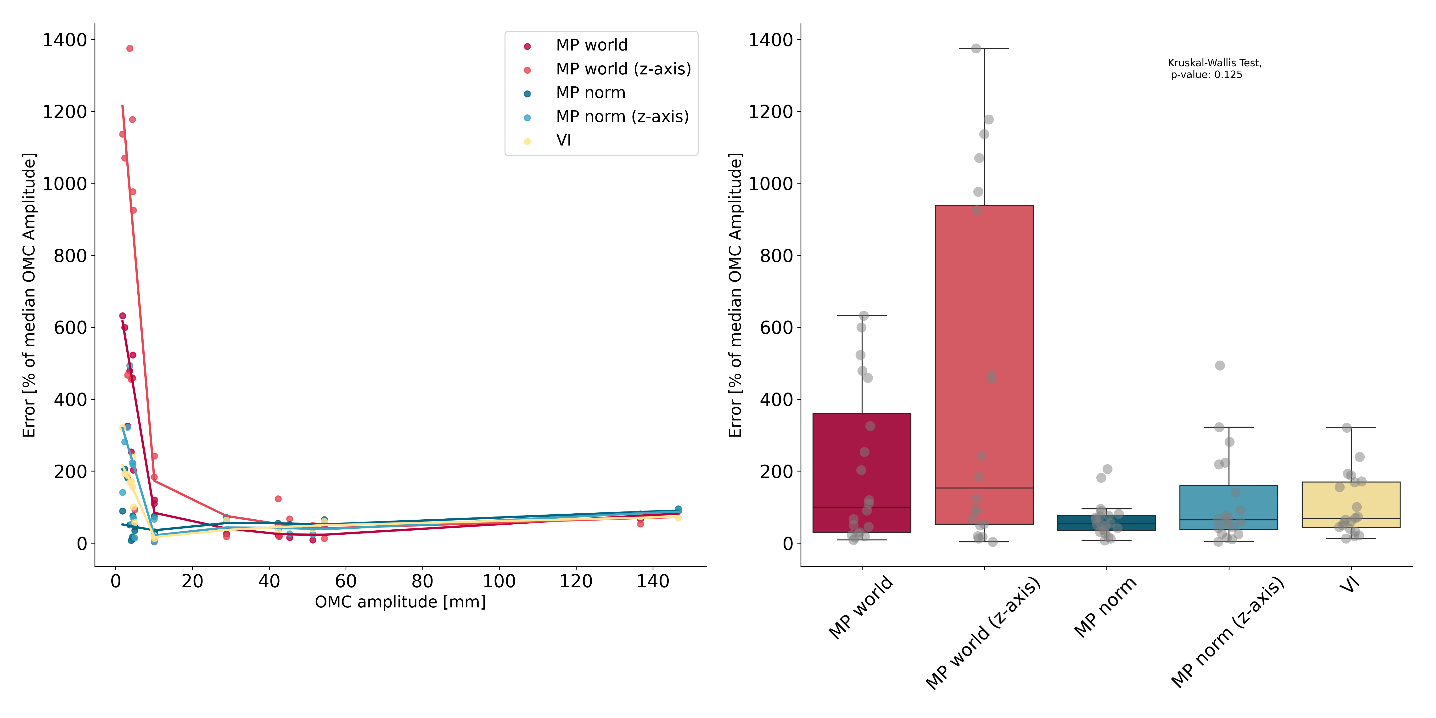


Supplementary Figure 5. The left scatter plot (A) shows the amplitude estimation error of the different methods expressed relative to OMC in % of the actual OMC amplitude and the OMC amplitude in millimeter. For tremor amplitudes of approximately <10 mm the amplitude estimation error and actual ground-truth amplitude converge and the relative error increases. (B) The bottom boxplots depict the relative amplitude estimation error across the different methods. There were not significant differences of the median estimation error, however the variability of MP world and MP world with z-axis was greater than the for the other methods (suppl. table 2). MP world = Mediapipe world landmarks, MP norm = Mediapipe normalized landmarks, VI = Vision (x/y).

Supplementary Table 2: Relative differences to OMC in % OMC of the estimated median amplitude

|  | Median | Minimum | IQR 25 | IQR 75 | Maximum |
| --- | --- | --- | --- | --- | --- |
| MP world | 100 | 9 | 29 | 359 | 632 |
| MP world (z-axis) | 153 | 3 | 52 | 938 | 1375 |
| MP norm | 53 | 7 | 34 | 77 | 206 |
| MP norm (z-axis) | 64 | 4 | 38 | 160 | 493 |
| VI | 68 | 12 | 43 | 169 | 321 |

| 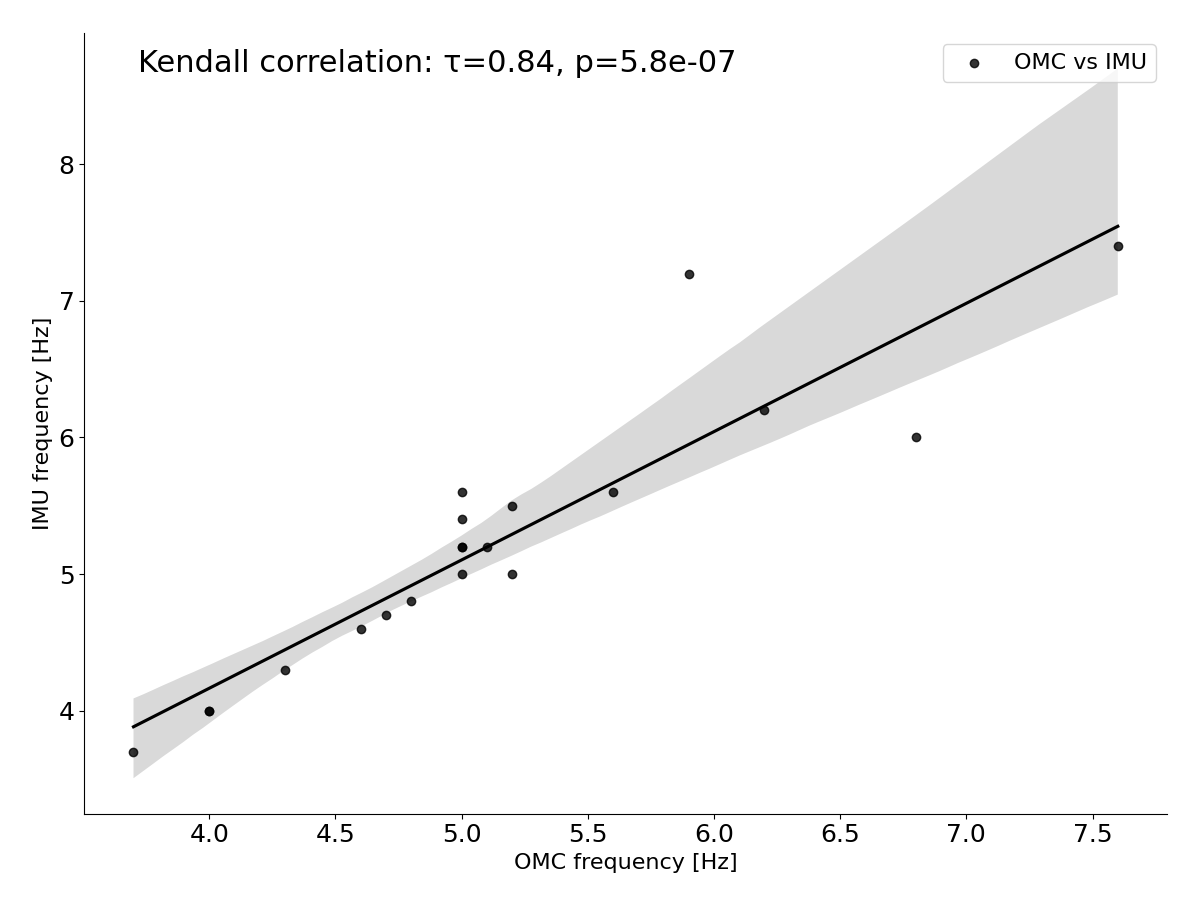  Supplementary Figure 6. The frequency estimation by OMC and IMU using the RAW pipeline showed only slight deviation between the identified peak frequency. |
| --- |

Supplementary Table 3: Absolute differences in Hz of detected peak frequencies.

|  | Median | Minimum | IQR 25 | IQR 75 | Maximum |
| --- | --- | --- | --- | --- | --- |
| MP vs OMC | 0.1 | 0 | 0 | 0.375 | 2.9 |
| MP vs IMU | 0.15 | 0 | 0 | 0.55 | 3.3 |
| VI vs OMC | 0.05 | 0 | 0 | 0.2 | 3.7 |
| VI vs IMU | 0.1 | 0 | 0 | 0.325 | 4.1 |

| 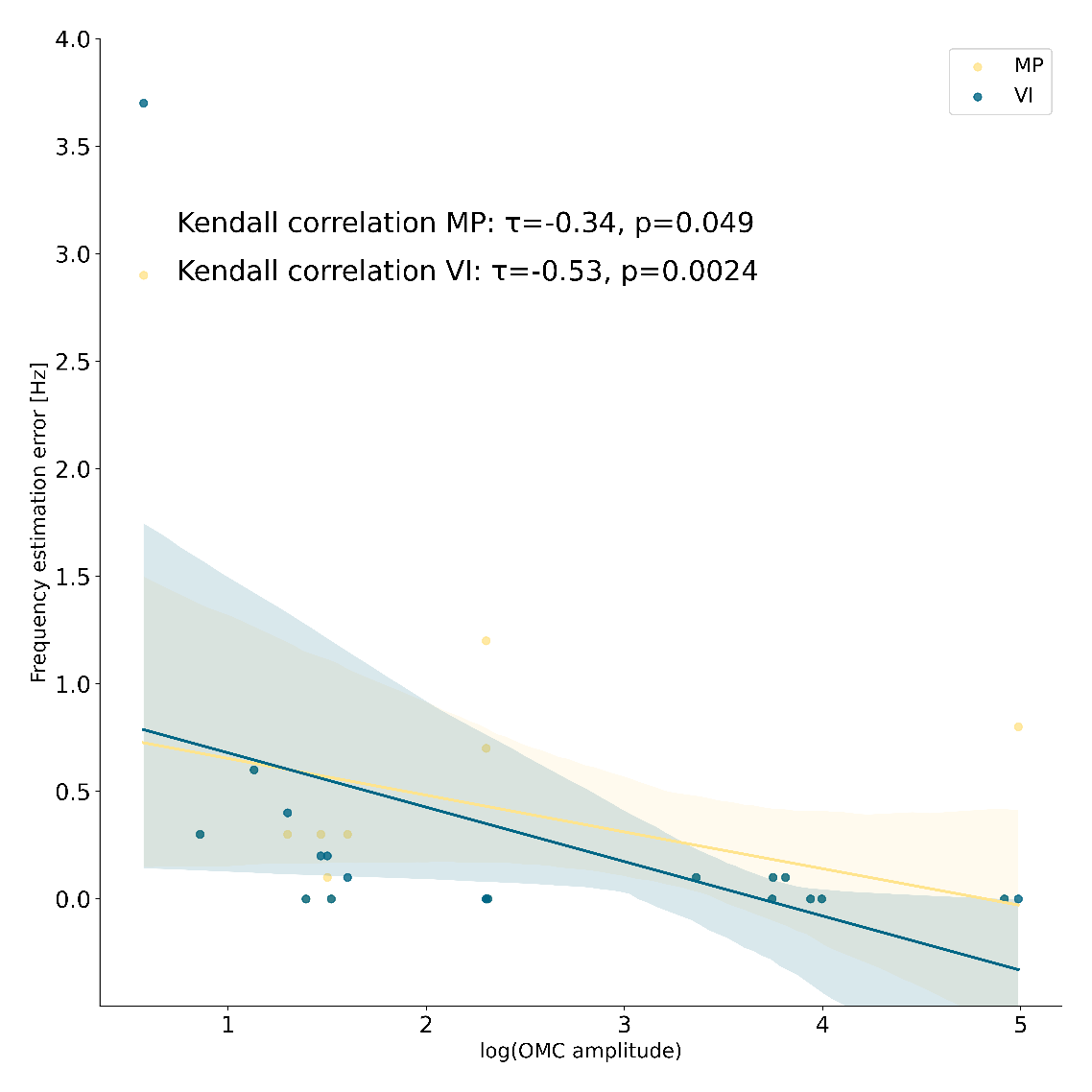  Supplementary Figure 7. These scatterplots show the logarithm of the OMC Amplitude in vs the frequency estimation error in Hz of MP and VI compared to the frequency detected by OMC. |
| --- |

Supplementary Table 4: Metric anchors boundaries of the estimated tremor amplitude using the TETRAS postural tremor of the upper extremities item for both postural and resting tremor (Elble 2016)

| Rating | Estimated tremor amplitude |
| --- | --- |
| 0 | No tremor |
| 0.5 | Uncertain if tremor is present |
| 1 | <0.5 cm |
| 1.5 | 0.5 - <1cm |
| 2 | 1 - <3cm |
| 2.5 | 3 - <5cm |
| 3 | 5 - < 10 cm |
| 3.5 | 10 - < 20 cm |
| 4 | >20 cm |
